# 4-Amino-2-phenoxy­pyrimidine

**DOI:** 10.1107/S1600536809026580

**Published:** 2009-07-15

**Authors:** Nasir Shah Bakhtiar, Zanariah Abdullah, Seik Weng Ng

**Affiliations:** a Department of Chemistry, University of Malaya, 50603 Kuala Lumpur, Malaysia

## Abstract

In the title compound, C_10_H_9_N_3_O, the organic rings linked to the ether O atom make a dihedral angle of 76.8 (1)° and the C—O—C angle is widened to 119.07 (15)°. In the crystal, adjacent mol­ecules are connected by an N—H⋯N hydrogen bond, generating a chain running parallel to the *b* axis. The crystal is a non-merohedral twin with a ratio of twin components of 0.508 (3):0.492 (3).

## Related literature

For 2-phenoxy­pyrimidine, see: Shah Bakhtiar *et al.* (2009 ▶). For the procedure to cope with twinned diffraction data, see: Spek (2003 ▶).
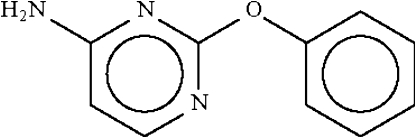


## Experimental

### 

#### Crystal data

C_10_H_9_N_3_O
*M* *_r_* = 187.20
Monoclinic, 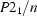

*a* = 8.8443 (3) Å
*b* = 12.1214 (3) Å
*c* = 9.0415 (2) Å
β = 96.751 (2)°
*V* = 962.58 (5) Å^3^
*Z* = 4
Mo *K*α radiation
μ = 0.09 mm^−1^
*T* = 120 K
0.40 × 0.20 × 0.10 mm

#### Data collection

Bruker SMART APEX diffractometer
Absorption correction: none
6375 measured reflections
2178 independent reflections
1694 reflections with *I* > 2σ(*I*)
*R*_int_ = 0.028

#### Refinement

*R*[*F* ^2^ > 2σ(*F* ^2^)] = 0.055
*wR*(*F* ^2^) = 0.163
*S* = 1.10
2178 reflections
136 parameters
2 restraints
H atoms treated by a mixture of independent and constrained refinement
Δρ_max_ = 0.43 e Å^−3^
Δρ_min_ = −0.32 e Å^−3^

### 

Data collection: *APEX2* (Bruker, 2008 ▶); cell refinement: *SAINT* (Bruker, 2008 ▶); data reduction: *SAINT*; program(s) used to solve structure: *SHELXS97* (Sheldrick, 2008 ▶); program(s) used to refine structure: *SHELXL97* (Sheldrick, 2008 ▶); molecular graphics: *X-SEED* (Barbour, 2001 ▶); software used to prepare material for publication: *publCIF* (Westrip, 2009 ▶).

## Supplementary Material

Crystal structure: contains datablocks global, I. DOI: 10.1107/S1600536809026580/bt2994sup1.cif

Structure factors: contains datablocks I. DOI: 10.1107/S1600536809026580/bt2994Isup2.hkl

Additional supplementary materials:  crystallographic information; 3D view; checkCIF report

## Figures and Tables

**Table 1:** Hydrogen-bond geometry (Å, °)

*D*—H⋯*A*	*D*—H	H⋯*A*	*D*⋯*A*	*D*—H⋯*A*
N3—H1⋯N1^i^	0.88 (1)	2.12 (1)	2.992 (2)	173 (2)

Symmetry code: (i) 
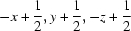
.

**Fig. 1.**
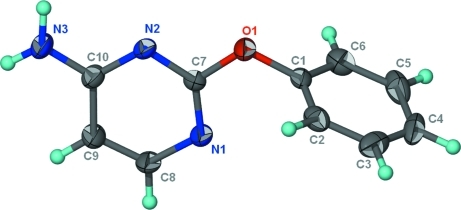
Anisotropic displacement ellipsoid plot (Barbour, 2001) of C10H9N3O at the 70% probability level; hydrogen atoms are drawn as spheres of arbitrary radius.

## supplementary crystallographic information

### Experimental

Phenol (1.88 g, 20 mmol) and sodium hydroxide (0.80 g, 20 mmol) were dissolved in water (50 ml) and to the solution was added 4-amino-2-chloropyridimidine (2.60 g, 20 mmol) dissolved in THF (50 ml). The mixture was heated for 4 h. Water was added and the organic phase was extracted with chloroform. The chloroform solution was dried over sodium sulfate; slow evaporation led to the formation of colorless crystals.

### Refinement

Carbon-bound H-atoms were placed in calculated positions (C—H 0.95 Å) and were included in the refinement in the riding model approximation, with *U*(H) set to 1.2*U*(C). The H atoms bonded to N were freely refined.

The crystal is a non-merohedral twin; the twin law as given by *PLATON* is (Spek, 2003) (-1 0 0, 0 - 1 0, 0.240 0 1); the refinement gave a  ratio of twin components of 0.508 (3)/0.492 (3).

### Crystal data

**Table:**

C_10_H_9_N_3_O	*F*(000) = 392
*M**_r_* = 187.20	*D*_x_ = 1.292 Mg m^−^^3^
Monoclinic, *P*2_1_/*n*	Mo *K*α radiation, λ = 0.71073 Å
Hall symbol: -P 2yn	Cell parameters from 2238 reflections
*a* = 8.8443 (3) Å	θ = 2.3–27.9°
*b* = 12.1214 (3) Å	µ = 0.09 mm^−^^1^
*c* = 9.0415 (2) Å	*T* = 120 K
β = 96.751 (2)°	Block, colorless
*V* = 962.58 (5) Å^3^	0.40 × 0.20 × 0.10 mm
*Z* = 4	

### Data collection

**Table:**

Bruker SMART APEX diffractometer	1694 reflections with *I* > 2σ(*I*)
Radiation source: fine-focus sealed tube	*R*_int_ = 0.028
graphite	θ_max_ = 27.5°, θ_min_ = 2.8°
ω scans	*h* = −10→11
6375 measured reflections	*k* = −15→15
2178 independent reflections	*l* = −10→11

### Refinement

**Table:**

Refinement on *F*^2^	Primary atom site location: structure-invariant direct methods
Least-squares matrix: full	Secondary atom site location: difference Fourier map
*R*[*F*^2^ > 2σ(*F*^2^)] = 0.055	Hydrogen site location: inferred from neighbouring sites
*wR*(*F*^2^) = 0.163	H atoms treated by a mixture of independent and constrained refinement
*S* = 1.10	*w* = 1/[σ^2^(*F*_o_^2^) + (0.0791*P*)^2^ + 0.3378*P*] where *P* = (*F*_o_^2^ + 2*F*_c_^2^)/3
2178 reflections	(Δ/σ)_max_ = 0.001
136 parameters	Δρ_max_ = 0.43 e Å^−^^3^
2 restraints	Δρ_min_ = −0.32 e Å^−^^3^

### Fractional atomic coordinates and isotropic or equivalent isotropic displacement parameters (Å^2^)

**Table:**

	*x*	*y*	*z*	*U*_iso_*/*U*_eq_	
O1	0.2422 (2)	0.48867 (12)	−0.00659 (16)	0.0317 (4)	
N1	0.2790 (2)	0.36872 (13)	0.19209 (18)	0.0235 (4)	
N2	0.2591 (2)	0.56448 (13)	0.21860 (17)	0.0213 (4)	
N3	0.2755 (3)	0.64646 (15)	0.4479 (2)	0.0348 (5)	
H1	0.252 (3)	0.7092 (13)	0.402 (2)	0.030 (7)*	
H2	0.284 (3)	0.641 (2)	0.5458 (11)	0.037 (7)*	
C1	0.2551 (3)	0.39941 (16)	−0.1029 (2)	0.0220 (5)	
C2	0.3964 (3)	0.35937 (18)	−0.1237 (2)	0.0264 (5)	
H2A	0.4857	0.3889	−0.0693	0.032*	
C3	0.4063 (3)	0.27495 (19)	−0.2257 (2)	0.0339 (6)	
H3	0.5030	0.2460	−0.2410	0.041*	
C4	0.2763 (4)	0.23278 (18)	−0.3052 (2)	0.0369 (7)	
H4	0.2836	0.1744	−0.3741	0.044*	
C5	0.1354 (3)	0.2756 (2)	−0.2843 (2)	0.0374 (6)	
H5	0.0461	0.2475	−0.3403	0.045*	
C6	0.1240 (3)	0.35942 (19)	−0.1819 (2)	0.0307 (5)	
H6	0.0274	0.3887	−0.1665	0.037*	
C7	0.2617 (2)	0.47102 (16)	0.1428 (2)	0.0197 (4)	
C8	0.2997 (3)	0.36125 (17)	0.3429 (2)	0.0283 (5)	
H8	0.3133	0.2900	0.3862	0.034*	
C9	0.3022 (3)	0.44951 (17)	0.4354 (2)	0.0296 (5)	
H9	0.3190	0.4413	0.5405	0.036*	
C10	0.2785 (3)	0.55438 (16)	0.3677 (2)	0.0236 (5)	

### Atomic displacement parameters (Å^2^)

**Table:**

	*U*^11^	*U*^22^	*U*^33^	*U*^12^	*U*^13^	*U*^23^
O1	0.0606 (12)	0.0159 (7)	0.0178 (7)	0.0050 (7)	0.0018 (7)	0.0013 (5)
N1	0.0341 (11)	0.0165 (8)	0.0197 (8)	0.0014 (7)	0.0032 (7)	0.0007 (6)
N2	0.0281 (10)	0.0149 (8)	0.0212 (8)	0.0028 (7)	0.0038 (7)	0.0003 (6)
N3	0.0665 (16)	0.0182 (9)	0.0204 (9)	0.0070 (9)	0.0083 (9)	−0.0008 (7)
C1	0.0351 (13)	0.0156 (9)	0.0151 (9)	0.0006 (8)	0.0018 (8)	0.0031 (7)
C2	0.0301 (12)	0.0255 (10)	0.0229 (10)	−0.0038 (9)	0.0005 (9)	0.0044 (8)
C3	0.0476 (15)	0.0264 (11)	0.0310 (11)	0.0084 (11)	0.0186 (11)	0.0062 (9)
C4	0.073 (2)	0.0193 (10)	0.0198 (10)	−0.0061 (12)	0.0127 (11)	−0.0026 (8)
C5	0.0511 (17)	0.0348 (12)	0.0238 (11)	−0.0161 (12)	−0.0058 (11)	0.0001 (9)
C6	0.0311 (13)	0.0319 (12)	0.0284 (11)	0.0006 (10)	0.0013 (10)	0.0036 (9)
C7	0.0217 (11)	0.0186 (9)	0.0185 (9)	0.0015 (8)	0.0012 (8)	0.0019 (7)
C8	0.0455 (14)	0.0174 (9)	0.0221 (10)	0.0044 (9)	0.0046 (9)	0.0046 (7)
C9	0.0482 (15)	0.0216 (10)	0.0194 (9)	0.0028 (10)	0.0057 (9)	0.0027 (7)
C10	0.0312 (12)	0.0181 (9)	0.0223 (9)	0.0008 (8)	0.0062 (9)	−0.0007 (7)

### Geometric parameters (Å, °)

**Table:**

O1—C7	1.359 (2)	C2—H2A	0.9500
O1—C1	1.402 (2)	C3—C4	1.380 (4)
N1—C7	1.320 (2)	C3—H3	0.9500
N1—C8	1.357 (3)	C4—C5	1.384 (4)
N2—C7	1.326 (2)	C4—H4	0.9500
N2—C10	1.344 (2)	C5—C6	1.386 (3)
N3—C10	1.333 (3)	C5—H5	0.9500
N3—H1	0.880 (10)	C6—H6	0.9500
N3—H2	0.882 (10)	C8—C9	1.356 (3)
C1—C2	1.375 (3)	C8—H8	0.9500
C1—C6	1.376 (3)	C9—C10	1.416 (3)
C2—C3	1.387 (3)	C9—H9	0.9500
			
C7—O1—C1	119.07 (15)	C4—C5—C6	120.2 (2)
C7—N1—C8	113.46 (17)	C4—C5—H5	119.9
C7—N2—C10	115.63 (16)	C6—C5—H5	119.9
C10—N3—H1	119.1 (16)	C1—C6—C5	118.8 (2)
C10—N3—H2	118.5 (18)	C1—C6—H6	120.6
H1—N3—H2	122 (2)	C5—C6—H6	120.6
C2—C1—C6	121.9 (2)	N1—C7—N2	129.54 (18)
C2—C1—O1	120.0 (2)	N1—C7—O1	118.65 (17)
C6—C1—O1	118.0 (2)	N2—C7—O1	111.80 (16)
C1—C2—C3	118.7 (2)	C9—C8—N1	123.86 (18)
C1—C2—H2A	120.6	C9—C8—H8	118.1
C3—C2—H2A	120.6	N1—C8—H8	118.1
C4—C3—C2	120.4 (2)	C8—C9—C10	116.79 (18)
C4—C3—H3	119.8	C8—C9—H9	121.6
C2—C3—H3	119.8	C10—C9—H9	121.6
C3—C4—C5	119.9 (2)	N3—C10—N2	117.46 (18)
C3—C4—H4	120.0	N3—C10—C9	121.85 (18)
C5—C4—H4	120.0	N2—C10—C9	120.69 (18)
			
C7—O1—C1—C2	−76.8 (2)	C8—N1—C7—O1	179.1 (2)
C7—O1—C1—C6	107.6 (2)	C10—N2—C7—N1	0.8 (3)
C6—C1—C2—C3	−1.1 (3)	C10—N2—C7—O1	−179.63 (19)
O1—C1—C2—C3	−176.53 (17)	C1—O1—C7—N1	−5.8 (3)
C1—C2—C3—C4	0.4 (3)	C1—O1—C7—N2	174.56 (18)
C2—C3—C4—C5	0.7 (3)	C7—N1—C8—C9	0.2 (4)
C3—C4—C5—C6	−1.2 (3)	N1—C8—C9—C10	1.3 (4)
C2—C1—C6—C5	0.6 (3)	C7—N2—C10—N3	−179.5 (2)
O1—C1—C6—C5	176.15 (18)	C7—N2—C10—C9	0.9 (3)
C4—C5—C6—C1	0.5 (3)	C8—C9—C10—N3	178.6 (2)
C8—N1—C7—N2	−1.3 (3)	C8—C9—C10—N2	−1.8 (4)

### Hydrogen-bond geometry (Å, °)

**Table:**

*D*—H···*A*	*D*—H	H···*A*	*D*···*A*	*D*—H···*A*
N3—H1···N1^i^	0.88 (1)	2.12 (1)	2.992 (2)	173 (2)

Symmetry codes: (i) −*x*+1/2, *y*+1/2, −*z*+1/2.
